# Digital Transformation in the Diagnostics and Therapy of Cardiovascular Diseases: Comprehensive Literature Review

**DOI:** 10.2196/44983

**Published:** 2023-08-30

**Authors:** Christopher Stremmel, Rüdiger Breitschwerdt

**Affiliations:** 1 Department of Cardiology, LMU University Hospital Munich Germany; 2 Wilhelm Büchner Hochschule, Mobile University of Technology Darmstadt Germany

**Keywords:** cardiovascular, digital medicine, telehealth, artificial intelligence, telemedicine, mobile phone, review

## Abstract

**Background:**

The digital transformation of our health care system has experienced a clear shift in the last few years due to political, medical, and technical innovations and reorganization. In particular, the cardiovascular field has undergone a significant change, with new broad perspectives in terms of optimized treatment strategies for patients nowadays.

**Objective:**

After a short historical introduction, this comprehensive literature review aimed to provide a detailed overview of the scientific evidence regarding digitalization in the diagnostics and therapy of cardiovascular diseases (CVDs).

**Methods:**

We performed an extensive literature search of the PubMed database and included all related articles that were published as of March 2022. Of the 3021 studies identified, 1639 (54.25%) studies were selected for a structured analysis and presentation (original articles: n=1273, 77.67%; reviews or comments: n=366, 22.33%). In addition to studies on CVDs in general, 829 studies could be assigned to a specific CVD with a diagnostic and therapeutic approach. For data presentation, all 829 publications were grouped into 6 categories of CVDs.

**Results:**

Evidence-based innovations in the cardiovascular field cover a wide medical spectrum, starting from the diagnosis of congenital heart diseases or arrhythmias and overoptimized workflows in the emergency care setting of acute myocardial infarction to telemedical care for patients having chronic diseases such as heart failure, coronary artery disease, or hypertension. The use of smartphones and wearables as well as the integration of artificial intelligence provides important tools for location-independent medical care and the prevention of adverse events.

**Conclusions:**

Digital transformation has opened up multiple new perspectives in the cardiovascular field, with rapidly expanding scientific evidence. Beyond important improvements in terms of patient care, these innovations are also capable of reducing costs for our health care system. In the next few years, digital transformation will continue to revolutionize the field of cardiovascular medicine and broaden our medical and scientific horizons.

## Introduction

### From Digitization to a Digital Transformation

Digitization is the conversion of analog information into a digital signal consisting of discrete values. Beyond the obvious advantage of storing data for the future, it also provides the potential for further IT processing. In general, the use of digitization to facilitate and optimize processes is referred to as digitalization. With the introduction of the first universally programmable computer by Konrad Zuse in 1941, a path was initiated that led us to an age at the end of the 20th century since when digital technologies have become omnipresent [1-4].

Over the years, the original term digitalization has expanded to a new strategic alignment of entire industries, as it nowadays also refers to the megatrend of digital transformation in all areas of life. Similar to how the Industrial Revolution shaped the transition from an agrarian to an industrial society 200 years ago, we are now experiencing a rapidly progressing digital revolution [2-4].

### Digital Medicine and Digital Health

Digitalization has become increasingly important in the field of medicine over the last 3 decades. The constantly growing volume of patient data makes the use of digital management systems inevitable. Redundant examinations must be avoided to ensure patient safety as well as from a health economics perspective. Data should be easily accessible in a digital patient file regardless of the location and should be provided to the attending physician upon patient consent. Moreover, there are innovative concepts in informatics, such as the implementation of artificial intelligence in the health care sector, that can decisively enrich the quality of medicine. Today, we are in the process of digital transformation, as digitalization has essentially transformed the entire medical sector as an industry.

Digital health, as a subdiscipline of digital medicine, describes a transformation toward patient-centered recordings of medical information including vital signs and simple diagnostics such as electrocardiograms (ECGs). It improves the security of supply and access to medical facilities, as it is separated from any local medical institution. A growing shortage of physicians in rural regions and restrictions due to the COVID-19 pandemic over the past 3 years have shown us how quickly critical medical care bottlenecks can occur. Telemedicine and digital remote diagnostics and treatments can provide enormous benefits in these situations [5].

### Smart Technologies and Lifestyle

The increasing integration of medical digital technology in everyday life in the form of digital health diaries, smartphones, smartwatches, and personal health monitoring systems has made the technical possibilities more tangible.

Even though smartphones have been in use since 1999, the launch of the first iPhone in 2007 triggered a noticeable change. Today, an estimated 4 billion people, or half of the world’s population, use a smartphone [6].

As a mobile minicomputer, the phone accompanies most people around the clock, and many of them can no longer imagine life without a smartphone. Specialized apps have been developed for a wide range of tasks including health care. Although these were purely informative in the first few years, there has been an increasing rethinking of how these data can be used in everyday patient care. As approximately 500 million people worldwide live with cardiovascular diseases (CVDs), it becomes obvious that smart technologies have enormous potential for prevention, diagnostics, and therapy monitoring [7].

A growing number of people continuously wear mobile minicomputers on their wrists (so-called wearables) to record activity data or vital signs. Prominent representatives of this class are smartwatches or fitness trackers. As with smartphones, the collected data are far more than just informative and have the potential to improve patient care. The most important parameters that can be recorded today are ECGs, heart rate, number of steps, oxygen saturation, and blood pressure or blood sugar values via special additional modules [8,9].

### CVD Diagnostics and Therapy

CVDs are highly interesting from the perspective of digital medicine because they have a very high lifetime prevalence, with approximately 500 million people affected worldwide, and they are responsible for approximately one-third of all deaths worldwide [7,10]. In fact, the World Health Organization assumes an even higher number of CVDs, as a significant proportion (potentially >50%) of people have not yet been diagnosed, especially in the case of arterial hypertension [7,10].

Cardiology involves the management of a broad spectrum of diseases ranging from chronic diseases such as heart failure or valvular heart diseases to highly acute pathologies such as acute myocardial infarction or cardiac arrhythmias. In addition, the technology of smartwatches and other so-called wearables consistently captures cardiovascular functional parameters such as heart rate, physical activity, or even blood pressure. For these reasons, great efforts have been made to make cardiovascular medicine as digital as possible to optimize patient care and treatment through innovative technologies.

The aim of this literature review was to present a comprehensive overview of the historical and technical development of digital technologies in relation to diagnostics and therapy for CVDs over the last few decades.

## Methods

### Literature Review

The aim of this study was to conduct a comprehensive literature review investigating digital transformation in the diagnostics and therapy of CVDs. The focus was on the direct application of digital technologies in patient care, whereas early phase technical innovations or IT solutions without direct clinical use were excluded. Therefore, we conducted an extensive literature search in the PubMed biomedical database, which provides ideal coverage of the publications of interest.

Our search strategy was based on two major components: (1) all studies that contained the terms “ehealth,” “digital health,” “artificial intelligence,” or “telemedicine” in the title or abstract were identified, and (2) the studies were narrowed down to the cardiovascular field by the simultaneous presence of the terms “cardiovascular” or “heart” in the title or abstract, resulting in the following search term: “((ehealth[Title/Abstract]) OR (digital health[Title/Abstract]) OR (artificial intelligence[Title/Abstract]) OR (telemedicine[Title/Abstract])) AND ((cardiovascular[Title/Abstract]) OR (heart[Title/Abstract])).” The choice of the above-mentioned search terms was the result of a survey of experienced cardiologists involved in digital cardiology at the University Hospital of Ludwig Maximilian University of Munich, Germany. Our search did not restrict any article types; therefore, review articles and comments were found among the results in addition to original articles. Furthermore, there was no thematic restriction within the cardiovascular field, and all studies up to the cutoff date (March 16, 2022) were included.

**Figure 1:**
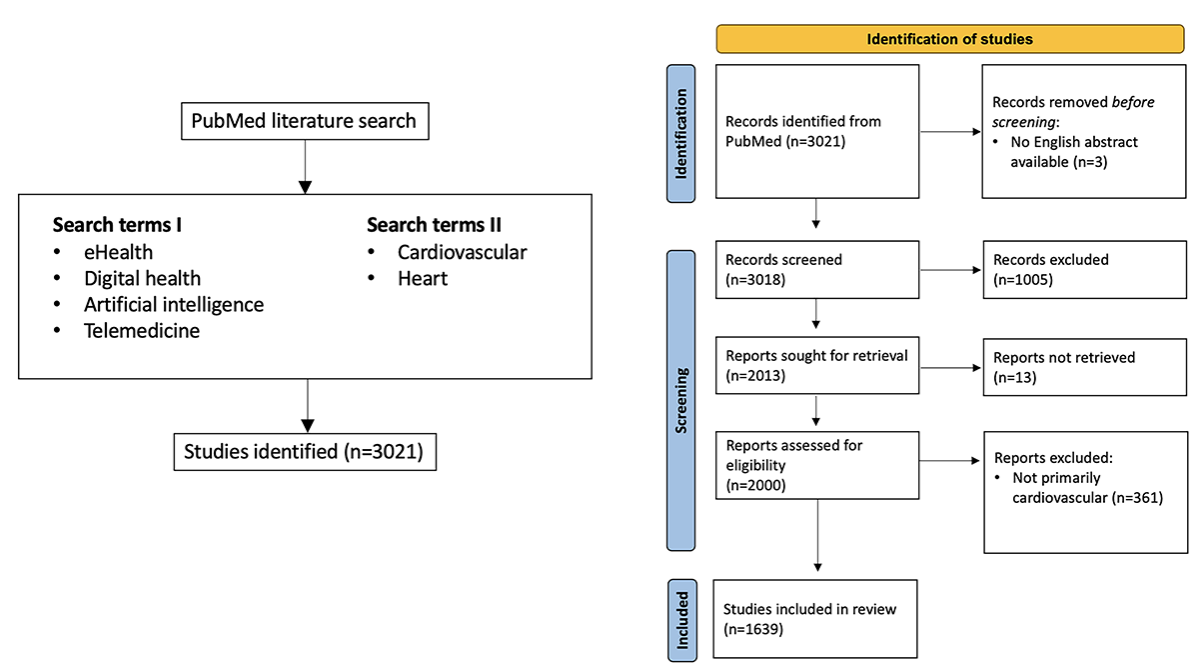
Literature search strategy and PRISMA (Preferred Reporting Items for Systematic Reviews and Meta-Analyses) flow diagram.

The review yielded 3021 studies, all of which were then screened and processed in a structured manner. Within this framework, 1018 (33.77%) of the 3021 studies were excluded because they were not directly related to digital technologies in medicine or because they could not be retrieved. In addition, 361 (11.95%) of the 3021 studies were excluded because they did not focus on CVDs, although they addressed digital technologies. Studies from interdisciplinary research areas such as diabetes mellitus as a major cardiovascular risk factor or dietary behavior as a preventive aspect were also excluded. The final analysis included 1639 studies of which 1273 (77.67%) were original articles and 366 (22.33%) were reviews or comments. In addition to studies on CVDs in general, 829 (50.58%) of the 1639 studies included in the final analysis could be assigned to a specific CVD with a diagnostic or therapeutic approach (Figure 1).

### Qualitative Structured Analysis

Qualitative analysis and structuring of the available sources were performed using ATLAS.ti software (version 22; ATLAS.ti Scientific Software Development GmbH). In the process, the so-called “codes” in the form of keywords were assigned to individual studies to reflect their content. The main keywords assigned here refer to the CVD pattern, type of digital application, and data processing methodology used in respective studies. In addition, the studies were separately labeled as dealing with new digital prototypes, thus indicating possible future applications.

**Figure 2:**
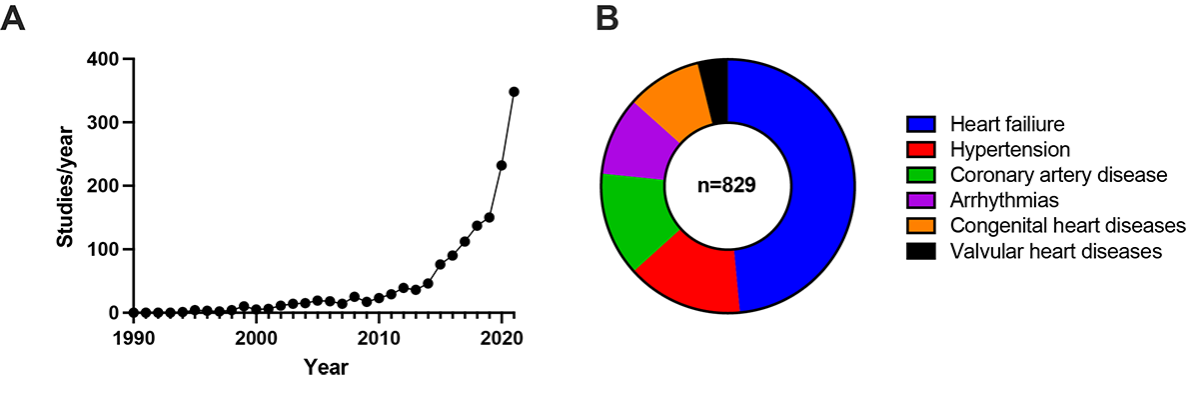
Publications in the field of digital cardiology. Depicted are (A) publication rates per year for studies on digital medicine in cardiology as well as (B) the respective area of cardiovascular medicine.

Building on the qualitative structuring described in the previous paragraph, content analysis was performed. All the 829 disease-specific publications were grouped by linking them to the following fields of cardiovascular medicine: (1) heart failure, (2) arterial hypertension, (3) coronary artery disease, (4) cardiac arrhythmias, (5) congenital heart diseases, and (6) valvular heart disease (Figure 2). In the *Results* section, a brief introduction to the disease itself and use cases of digital medicine are presented and underlined by the scientific data. For this purpose, the results of the screened studies are summarized. In the *Discussion* section, possible future projects and perspectives are outlined.

For visualization, we chose the evaluation of word frequencies, following established concepts of qualitative reporting [11-13]. As heart failure is the most extensively investigated condition, multiple randomized controlled trials have been published. A summary of the results is presented in Multimedia Appendix 1 [14-41] according to PRISMA (Preferred Reporting Items for Systematic Reviews and Meta-Analyses) guidelines.

## Results

### CVDs in Digital Medicine

A total of 829 studies in the field of digital medicine could be assigned to a specific CVD, with subsequent grouping into 6 major categories (Table 1 and Figure 2). All major areas of cardiology are covered by these studies, leaving no significant thematic gap between the prevalence of CVD patterns and digital implementation. Of note, the focus of research has shifted over the decades; whereas the focus was mainly concentrated on congenital heart defects and valvular heart diseases in the early days of cardiovascular digital medicine in the 1990s, current emphasis is on chronic diseases such as heart failure, coronary artery disease, and arterial hypertension. Another aspect of cardiovascular digital research focuses on the diagnosis of cardiac arrhythmias, which is certainly due to the growing use of wearables (Table 1).

**Table 1:** Grouping of digital cardiovascular research in the last decades (n=829).

	Published studies, n (%)			
	1990s (n=16)	2000s (n=87)	2010s (n=376)	2020s (n=350)
Heart failure	2 (12.5)	32 (36.8)	204 (54.3)	164 (46.9)
Hypertension	0 (0)	15 (17.2)	51 (13.6)	57 (16.3)
Coronary artery disease	1 (6.3)	10 (11.5)	48 (12.8)	51 (14.6)
Arrhythmias	0 (0)	2 (2.3)	33 (8.8)	48 (13.7)
Congenital heart diseases	11 (68.8)	24 (27.6)	27 (7.2)	17 (4.9)
Valvular heart diseases	2 (12.5)	4 (4.6)	13 (3.5)	13 (3.7)

In the following sections, individual cardiovascular clinical features are briefly introduced. This is followed by a concise description of the current diagnostics and therapy. On the basis of available evidence, digital health concepts are presented and critically evaluated, and potential future perspectives are highlighted.

### Heart Failure

Heart failure is defined as the inability of the heart to provide sufficient blood flow through the body, that is, cardiac output. This is usually due to reduced cardiac contractility, namely, systolic heart failure. The underlying pathomechanism is mostly lack of oxygen supply due to coronary artery disease [42]. Ischemic heart disease including heart failure accounts for almost 10 million deaths per year, making it the leading cause of death worldwide [10]. In the early stages, the 1-year mortality rate is <10% per year; it rises to up to 50% per year with disease progression and is thus far higher than that for most cancers. Heart failure is a chronic disease that requires consistent long-term therapy. In addition to the treatment of triggering underlying diseases, such as high blood pressure, there are defined therapy recommendations that have a clear focus on drug treatment.

Heart failure is one of the most important diseases in terms of health economics, and it has affected the lives of about 70 million people worldwide for many years [7,10]. It is not surprising that heart failure is the most intensively investigated condition in terms of digitalization in the cardiovascular field (Table 1 and Multimedia Appendix 1). A total of 402 studies, including 289 (71.9%) original articles and 113 (28.1%) reviews or comments, focused on digital options in diagnostics and therapy. Looking at the 10 most frequently used words in the titles of these publications, it is evident that telemedical management in the home environment has a strong focus (Textbox 1).

**Title words from studies on digital cardiology. Depicted are the 10 most frequently used words from titles of studies on indicated cardiovascular research areas in descending order.**

**Heart failure**

- Heart
- Failure
- Patients
- Chronic
- Management
- Care
- Telemedicine
- Study
- Monitoring
- Health

**Hypertension**

- Pressure
- Blood
- Hypertension
- Telemedicine
- Health
- Management
- Monitoring
- Patients
- Study
- Based

**Coronary artery disease**

- Disease
- Patients
- Health
- Coronary
- Heart
- Based
- Cardiovascular
- Trial
- Telemedicine
- Chronic

**Arrhythmias**

- Atrial
- Fibrillation
- Cardiac
- Smart
- Study
- Heart
- Health
- Monitoring
- Detection
- Patients

**Congenital heart diseases**

- Heart
- Congenital
- Disease
- Telemedicine
- Pediatric
- Fetal
- Remote
- Cardiology
- Diagnosis
- Health

**Valvular heart diseases**

- Heart
- Sound
- Telemedicine
- Auscultation
- Monitoring
- Stethoscope
- Development
- Study
- Based
- Cardiac

Living with a diagnosis of heart failure requires consistent use of prescribed medications over many years. Simultaneously, changes in vital signs, blood values, and cardiac function must be monitored regularly. As it is known that the maximum tolerated dose ensures the longest survival, a fine and regular adjustment of the current therapy is necessary. Furthermore, it is essential to check one’s body weight regularly, as this can indicate water retention in case of decompensated heart failure before more pronounced symptoms such as shortness of breath or a decrease in physical performance occur. Telemedicine offers excellent opportunities to provide urgently needed care, especially in medically underserved areas or, as recently, during the COVID-19 pandemic [43,44].

Studies that investigated telephone and telemedical care systems in an outpatient setting were able to demonstrate a significant reduction in hospitalization rates, whereas a trend toward reduced mortality did not reach statistical significance [14,45,46]. Large, randomized follow-up studies such as *Telemonitoring to Improve Heart Failure Outcomes* (Tele-HF), *Telemedical Interventional Monitoring in Heart Failure* (TIM-HF), or *Baroreflex Activation Therapy for Heart Failure* (BEAT-HF) could not confirm the reduction in hospitalization rates. The authors concluded that patient selection may have a crucial impact on success and that it needs to be critically evaluated in the future [15,16]. Meta-analyses on this topic have identified a slight reduction in all-cause mortality and heart failure–associated hospitalizations [47-49]. Recent trials that have optimized their concepts in terms of technology and patient selection, such as TIM-HF2 or *A New Model of Medical Care With Use of Modern Methods of Non-invasive Clinical Assessment and Telemedicine in Patients With Heart Failure* (AMULET), showed a reduction in hospitalization rates as well as all-cause mortality (TIM-HF2) and cardiovascular mortality (AMULET) [17,18]. This has led the European Cardiology Society to provide telemedical procedures for the treatment of heart failure, with a recommendation grade IIB in its guidelines [42] (Multimedia Appendix 1).

It is clear that in the field of heart failure, the focus is less on diagnostics and more on optimized monitoring of therapy using modern digital tools. Most studies on digital technologies in heart failure use telemedical applications to care for patients in their home environment. This reduces on-site visits and allows for earlier discharge of patients from the hospital, with telemedical support during the transition phase. Several studies have demonstrated a significant reduction in the 30-day rehospitalization rate from 24% to 17% to 18% [50,51]. In-depth heart failure education is provided to patients as part of their respective telemedicine programs [52,53]. In addition, telemedical services facilitate communication and involve psychological factors such as increased social attention, which should not be neglected [54,55]. Vital signs and body weight can be recorded autonomously via wearables or similar devices that transmit data to the attending physician or heart failure nurse [56]. Moreover, individual medications can be evaluated and adjusted using telemedicine applications [19,20,57]. Current experimental studies have attempted to integrate telemedicine ultrasound diagnostics by trained nonphysician staff or the patients themselves [58,59]. In addition to these conventional digital methods, artificial intelligence algorithms that have the potential to automate therapy adjustments based on transmitted data are being developed [60,61].

In patients with special pacemakers for cardiac resynchronization or those with implanted cardiac defibrillators, further data can be collected via remote monitoring systems without direct physician-patient contact. These include physical activity and heart rate profiles, detection of possible arrhythmias, and impedance-guided detection of water balance in the body [62]. Studies on this monitoring mechanism have been controversial; whereas SENSE-HF, OptiLinkHF, and *Remote Management of Heart Failure Using Implanted Electronic Devices *(REM-HF) failed to show a clear benefit [21,22,63], a reduction in hospitalizations was demonstrated in the *Evolution of Management Strategies of Heart Failure Patients With Implantable Defibrillators* (EVOLVO) and *Influence of Home Monitoring on Mortality and Morbidity in Heart Failure Patients With Impaired Left Ventricular Function* (IN-TIME) studies [23,24]. Regardless of the latter discussion, the establishment of remote monitoring allows for cost savings during routine follow-ups [25,64] (Multimedia Appendix 1).

In addition to these implanted devices with primarily therapeutic intention, there are also purely diagnostic monitoring tools such as loop recorders for the detection of arrhythmias or devices for the real-time measurement of pulmonary arterial pressure as a surrogate for hemodynamics in patients with heart failure [65]. For example, the *CardioMEMS Heart Sensor Allows Monitoring of Pressure to Improve Outcomes in New York Heart Association Class III Heart Failure Patients* (CHAMPION) trial for the CardioMEMS system demonstrated that the pulmonary arterial pressure could be lowered, and cardiac symptoms and hospitalization rates were reduced by using this monitoring device [26]. A technologically comparable approach to measuring pulmonary arterial pressure in heart failure is currently being investigated using the Cordella system, although the final study results are still pending [66]. Possible approaches to these systems are not only the early detection of heart failure decompensations but also a more differentiated therapy control including an automated adjustment of left ventricular assist devices [67].

Patients with heart failure clearly benefit from adapted fitness programs such as those offered in rehabilitation clinics or special cardiac training groups, despite their limited exercise capacity. Given the increased baseline risk, monitoring vital signs, with subsequent transmission to the attending physician or an intelligent system, offers tremendous opportunities for improved exercise planning. Other tools provide specific exercise recommendations and guidance for patients with heart failure via web platforms or smartphones. These telemedicine fitness offerings, especially those for rehabilitation, have a positive impact on patient compliance and quality of life and are noninferior to on-site programs in their effectiveness [68-70].

Overall, digital technologies have become a major tool in guiding patients with heart failure and offer enormous potential for optimized therapy adjustments while substantially reducing planned and unplanned medical consultations in inpatient and outpatient settings. Although the use of digital technologies is associated with additional costs, numerous studies have shown that these systems perform positively in the overall cost balance and reduce financial burdens for health care systems by 10% to 35% while increasing patient comfort [71,72].

### Arterial Hypertension

Arterial hypertension is defined as blood pressure >140 mm Hg systolic or >90 mm Hg diastolic blood pressure as measured in the physician’s office. The estimated prevalence according to the World Health Organization is approximately 1.3 billion people worldwide, whereas more than half of them have not been diagnosed yet [73].

Depending on the severity of hypertension and individual cardiovascular risk factors, the initial treatment strategies for mild forms of hypertension can mainly focus on lifestyle changes. These include reduced stress, regular physical activity, and a healthy diet. If these measures fail, drug therapy is initiated according to the current guideline recommendations. For rare cases in which a secondary form of hypertension is present, the first-line treatment involves managing the underlying causes [74].

Digital medicine finds its application in both diagnostics and therapy of hypertension in 123 studies (including 26 reviews or comments). Keywords in the titles of these studies predominantly describe the remote monitoring of blood pressure by telemedicine systems using wearables (Textbox 1).

Autonomously performed outpatient blood pressure measurements, which are forwarded to a physician using a digital system, can be important for identifying and treating patients with hypertension. Various factors play a decisive role here: blood pressure measurements should be as uncomplicated and accurate as possible. To this end, various systems that allow measurements via smartphone-connected devices have been validated [75]. Although these systems used to be predominantly cuff based, modern alternatives do not require the classic pressure cuff. The common basis for this is photoplethysmography in which light is registered differently depending on the blood flow by a photosensor in the form of a pulse wave. This is performed either via a smartphone camera or contactless via webcams and comparable devices. In combination with intelligent algorithms, blood pressure can be calculated accurately to approximately 5±8 mm Hg either from the waveform or the pulse transit time (rarely also oscillometrically or via the detection of heart sounds) [76]. Artificial intelligence is used to further increase the reliability and measurement accuracy [76,77]. Although current cuff-free systems have not yet found widespread use and certainly need to be optimized, there is great potential for identifying and monitoring patients with hypertension or detecting fluctuations in blood pressure over the course of the day using smartphone-based self-measurement systems [78]. The latest devices have incorporated pressure sensors in special textiles to record blood pressure changes, and they are barely noticeable [79,80]. The technology of continuous autonomous measurements allows the evaluation of blood pressure variability and therefore helps further specify the individual cardiovascular risk [81,82].

Blood pressure monitoring also plays a crucial role in hospitals. In specialized centers or intensive care settings, blood pressure is automatically recorded at specific intervals and sent to a central monitoring unit. As an alternative to conventional monitoring systems, institutions first started to use cost-effective wearables to monitor selected patients [83].

In addition to the diagnosis of hypertension, therapy planning and management are also of major importance. Especially in primary care, blood pressure therapy is a significant topic, and it is not always easy to identify the best therapy according to current recommendations for the individual patient. The first pioneering study used intelligent decision support systems to optimize guideline-based therapy, which was regarded as helpful by most treating physicians [84]. Continuous adjustment of blood pressure medication to recorded values and monitoring of possible side effects require close medical supervision. However, personal visits might become dispensable if blood pressure measurements are performed autonomously, followed by digital adjustments of the respective medication. This is particularly appealing in medically underserved areas [85,86]. Telemedical systems for blood pressure monitoring and therapy adjustment have also proven useful in the transition from inpatient care to outpatient therapy [87].

Several randomized controlled trials have shown inconsistent data on the success of telemedicine interventions for the treatment of arterial hypertension. The type of intervention ranges from simple text messages for educational purposes, over internet platforms and smartphone apps, to detailed telemedical consultations in video conferences with physicians [88-90]. Depending on the type of intervention, meta-analyses have shown an additional reduction in systolic blood pressure of up to 4 mm Hg compared with conventional therapy. Interestingly, the increased interactivity of telemedicine care was associated with increased therapeutic success [91,92].

The high prevalence of hypertension and associated outpatient consultations or hospital admissions suggests that telemedicine systems can save costs. Although there is a tendency for an overall reduction in costs, a conclusion is still missing and needs to be addressed in future studies [93].

### Coronary Artery Disease

Coronary artery disease involves the pathology of the heart vessels (coronaries) that ultimately impairs circulation and thus leads to a potential reduction in the supply of oxygen to the heart muscle as is typically found in myocardial infarction. The causes of the development of coronary heart disease are high blood pressure, elevated blood lipid levels (cholesterol), smoking, diabetes mellitus, and a family history of associated diseases. In industrialized nations, coronary heart disease is the leading cause of death (responsible for approximately 20% of all cases) and ranks among the top 3 worldwide [10]. Currently, more than 200 million people worldwide are affected by ischemic heart disease [7].

The mainstay of treatment for coronary heart disease is to reduce the risk of CVDs. If these methods fail and the coronary vessel is narrowed to an extent that significantly limits blood flow, it is reopened by percutaneous coronary intervention. For selected patients with the most severe forms of coronary artery disease, bypass surgery must also be considered to ensure coronary perfusion [94].

For these reasons, coronary artery disease has been a major focus in the field of digital medicine, with 110 publications (including 42 reviews or comments) investigating this domain. Thematic focuses that are based on the keywords of the study titles concern both acute and chronic telemedical care of coronary artery disease for primary and secondary prevention (Textbox 1).

Diagnostic apps offer a broad screening potential and the chance to identify high-risk patients [95]. Cardiovascular risk factors such as immobility, elevated blood pressure, or obesity can be easily detected by smartphones and associated devices to generate an awareness of the potential risk. With modern algorithms, in addition to heart rate variability, pulse wave velocity can be determined as an early marker of cardiovascular changes using photoplethysmography technology [96-98].

Even more important, however, is the role of digital diagnostics through wearable devices and telemedicine in secondary prevention. Patients can use smart technologies to monitor their health status based on vital signs, obtain information about their illness, manage and adjust their digital medication plans, and maintain regular web-based communication with their responsible physicians [99,100]. At the same time, there are programs that enable patients to autonomously manage their disease and obtain relevant information using apps or the web [101]. In particular, the transition from inpatient to outpatient care can also be significantly improved by telemedicine care, and any uncertainties that may arise early can be quickly eliminated [102,103]. Medical care is improved while saving costs by reduced rehospitalizations at the same time [104]. This also applies to rehabilitation therapy, where telemedicine measures allow the monitoring of vital signs during physical activity. Intensive engagement with the disease can be promoted, facilitating the transition to a normal everyday life. Several meta-analyses have demonstrated significant benefits of the use of telemedicine systems in cardiac rehabilitation, particularly in terms of physical performance, quality of life, and disease education [105-107].

Time is often critical when acute cardiac symptoms occur. Typical ECG changes (ST-segment or T-wave alterations) that occur in life-threatening myocardial infarction can be detected very quickly. Artificial intelligence algorithms are applied to accelerate and simplify this crucial diagnostic procedure, which can even be performed when no physician is present [108-110]. In this way, we can obtain highly relevant clinical information, and the necessary therapeutic steps can be initiated as early as possible. This applies not only to the transmission of data collected by the patient through smartphones or other wearables but also to ECG transmissions in the context of emergency medical services. Typical ECG changes in myocardial infarction and arrhythmias can be recorded, or vital signs such as blood pressure or oxygen saturation can be transmitted [9]. A meta-analysis showed that telemedical ECG transmission reduces the door-to-balloon time (time from hospital admission to reopening of the infarct vessel) by up to 30 minutes, which reduces mortality in the short- and long-term [111,112]. Especially in rural areas, where medical services are often less accessible, the use of telemedical systems can offer an enormous advantage [113]. Even during the COVID-19 pandemic, digital medicine filled important gaps in patient care, as many patients avoided medical contact whenever possible [114]. Beyond the obvious benefits of optimized care for coronary artery disease, telemedicine systems offer a relevant saving potential in terms of health economics [115].

### Arrhythmias

Under physiological conditions, cardiac action is triggered by the sinus node. From there, the cardiac action spreads through the atrial tissue to the atrioventricular node, from where it is transmitted to the ventricle. When this system is disturbed or in cases of automaticity, fibrosis, or scarring, cardiac arrhythmias occur, originating in either the atrium or the ventricle.

To detect cardiac arrhythmias, an ECG must be recorded whenever possible. If an episode of arrhythmia was not documented, a long-term ECG over 24 to 48 hours can be performed or the patient can be connected to permanent monitoring devices in the inpatient setting. If a cardiac arrhythmia can be confirmed, the therapy depends entirely on the type of arrhythmia. In the context of this review, we have limited ourselves to atrial fibrillation because other forms are rarely covered by studies in the field of digital cardiology.

Atrial fibrillation requires anticoagulation therapy to prevent the development of stroke. In addition, a strategy of rate control can be pursued, in which one accepts atrial fibrillation and uses only β-blocker therapy to counteract periods of rapid cardiac action. An alternative therapy goal is the conversion to normal sinus rhythm, the so-called rhythm control strategy. This can be performed by either electrical cardioversion or cardiac ablation (pulmonary vein isolation) [116].

Digital medicine offers far-reaching possibilities for the diagnosis of cardiac arrhythmia. The steady availability of spontaneous ECG recordings through smartwatches and other wearables has significantly facilitated the detection of rare rhythm events or assignment of symptoms to ECG images. A total of 83 publications, including 13 reviews or comments, addressed the applications of digital medicine in cardiac arrhythmias. Keywords from these papers map the monitoring and detection of arrhythmias, especially atrial fibrillation, by wearables as priority topics (Textbox 1).

The focus of digital applications in relation to cardiac arrhythmias is diagnostics. The current standard for the detection of arrhythmias is long-term ECG recordings or, in selected cases, the implantation of a loop recorder. Typical indications are unexplained syncope, the identification of atrial fibrillation in patients after (cryptogenic) stroke, or arrhythmias of any kind in the phase after an acute myocardial infarction [117]. However, today’s technology has set a path for significant change in the near future. Modern wearables offer recordings of cardiac rhythm mainly by photoplethysmography as well as ECGs with high resolution [118,119]. Using special algorithms based on heart rate variability and artificial intelligence, atrial fibrillation can be detected automatically in many cases, reaching a positive predictive value of ≥90% [120-122]. Large screening examinations for congenital arrhythmias or for monitoring of cardiomyopathies are feasible via telemedicine devices [123,124]. In addition, recent technical innovations have enabled rhythm diagnostics via special electrodes in shirts [125].

Patients with implanted pacemakers or defibrillators require regular follow-up. To reduce costs and increase patient comfort, this can be performed at least partially via remote monitoring [25]. Furthermore, it allows for intensified monitoring and earlier detection of possible malfunctions or cardiac arrhythmias.

In the therapeutic field, the applications of digital medicine for cardiac arrhythmias are still limited. First, there is certainly frequency monitoring in patients with atrial fibrillation [126]. Particularly in medically underserved areas or during the COVID-19 pandemic, digital procedures were able to ensure guideline-compliant patient care, which also led to an overall increase in the quality of life of affected patients [127]. In addition, there are now some smartphone apps that remind patients to take their anticoagulation therapy to prevent stroke, provide information about the therapy, and in some cases even provide specific recommendations for dose adjustments [128]. There are also approaches to smart medication lists beyond anticoagulation, which can improve both patient comfort and the medical treatment itself [129]. Similar to other CVDs, the transition from inpatient care during ablation treatment to outpatient care for patients with atrial fibrillation can be improved by telemedicine techniques: patients gain quality of life, recurrences are detected earlier, and overall, there is an increase in physical performance [130].

In the future, the detection of cardiac arrhythmias using smartwatches and other wearables will become more important. However, when detecting atrial fibrillation via optical sensors (photoplethysmography), as they are used in the Apple Watch, for example, we should always keep in mind that false alarms may occur and demand diagnostic confirmation via ECG recordings [122]. Nevertheless, the wide applicability offers a great advantage in arrhythmia detection that cannot be achieved by alternative methods [121,131].

### Congenital Heart Diseases

Almost 1% of newborns in industrialized countries have congenital heart defects. A basic distinction is made between cyanotic (blue skin in the presence of hypoxia) and acyanotic heart defects. The most common pathologies include defects in the ventricular or atrial septum (acyanotic), tetralogy of Fallot (cyanotic), and patent ductus arteriosus (acyanotic).

The most relevant cardiac defects are diagnosed during the prenatal examinations. However, it also happens that individual defects are detected only during birth. The essential examination procedures for the diagnosis of cardiac defects include auscultation of heart murmurs and echocardiography to confirm the diagnosis. Depending on the anatomical characteristics and severity of the defect, most corrections are performed via cardiac surgery or interventional procedures by the treating pediatric cardiologists. The only exception is patent ductus arteriosus, which can usually be closed using targeted drug therapy.

Diagnosis of congenital heart defects was the pioneering area of digital medicine in the field of cardiology (Table 1). Initial work on this topic was published in the early 1990s and totaled 79, including 12 reviews or comments [132]. Keywords from these publications focused on the telemedicine diagnosis of congenital heart defects using ultrasound (Textbox 1).

Timely diagnosis of congenital heart defects is crucial for the treatment of affected individuals. Therefore, high medical standards and a wealth of experience are of particular importance. Especially in smaller hospitals, in rural areas, or during the COVID-19 pandemic, bottlenecks in care can be compensated for by specialized physicians via telemedicine consultations or using artificial intelligence approaches [133,134]. Although the transmission of auscultation findings characterized the initial phase of digital cardiology, telemedicine specialist–guided ultrasound examinations dominate the field today [132,135,136]. This ensures high-level diagnostics regardless of the location, and, at the same time, training is provided to physicians who have less experience with relevant clinical pictures [137,138]. These modern approaches can not only reduce severe disease progression with long hospital stays by about 50% but also facilitate the early detection of cardiac defects, allowing significant cost savings [139].

Patients with congenital heart defects often require special care throughout their life. This is especially true in stressful situations such as surgery and physical activities. Telemedicine has enabled a unique network in outpatient care; affected persons can be closely monitored via wearables in their home environment and always have the option of obtaining further information or consulting a physician directly [140]. Initial investigations of home care suggested that the mortality of patients with congenital heart defects can be slightly reduced [141,142]. In addition, telemedicine in combination with wearables makes it possible to provide patients with individual training programs, thus increasing not only performance but also quality of life under controlled conditions [143,144].

### Valvular Heart Diseases

Heart valve diseases are defects in the closing or opening functions of at least one of the 4 heart valves. Indicators of potential heart valve defects can be obtained from auscultation using a stethoscope. The definite diagnosis is confirmed by ultrasound examination of the heart, which also allows for severity quantification. Valvular heart diseases are usually treated conservatively until they reach a relevant degree of severity. Therefore, patients with valvular defects need to be evaluated regularly with respect to exercise capacity, vital signs, laboratory parameters, and echocardiography findings to detect possible deterioration over time. Once the relevant severity of the defect is reached, younger patients are usually treated surgically, and older patients are usually treated by an interventional approach in the cardiac catheterization laboratory [145].

Acquired valvular heart diseases play important roles in everyday medical practice. A total of 32 publications, including 4 reviews, investigated digital technologies in this field. Similar to keywords related to congenital heart defects, the keywords focus on telemedical remote diagnostics using echocardiography (Textbox 1).

Beyond technical innovations in the field of image acquisition and processing, telemedicine remote diagnosis of echocardiography and artificial intelligence algorithms for automated analyses have yielded promising results [146-148]. In vitro model experiments have used special flow sensors in the ascending aorta; via continuous measurement techniques, these sensors were able to independently indicate the development of valvular heart diseases that require treatment [149]. Although this method is still far from use in humans, it offers insights into future diagnostic potential. In the context of the COVID-19 pandemic, in medically underserved areas, or to increase the quality of care in emergency departments, the use of digital technology in ultrasound examinations has experienced a significant upswing [150,151]. In parallel with the increasing use of remote telemedicine diagnostics by consulting experts, robotic systems have been tested to perform echocardiography [152,153]. Technological innovations such as automated acquisition of myocardial stiffness, among other measures of diastolic function, are being tested and show promising initial results [154].

The therapeutic applications of digital medicine in valvular heart diseases are limited. Nevertheless, the application of intelligent decision support systems seems to gain increasing clinical importance. In addition to technical data and guideline recommendations, past experience and patient preferences can be incorporated [155].

## Discussion

### Overview

Digital medicine in diagnostics and therapy for CVDs has experienced considerable upswing, particularly in the last 10 to 15 years. Political measures, such as adapted legislation, and special funding programs have laid a significant foundation for medical digitalization. In addition, there is a growing affinity among the population for innovative technical solutions and a more differentiated view of data protection concerns.

Beyond the structural conditions described in the previous paragraph, external circumstances have provided an additional boost to digital medicine. These factors include the shortage of physicians in rural areas and the COVID-19 pandemic during the last 3 years. Finally, the increased use of digital medicine is attributed to the advancing technology with the widespread use of smartphones and other wearables as well as the exchange of information via the internet and video conferences as part of our everyday lives [156]. In the medical sector, in particular, digitalization is rapidly expanding, even if the interoperability of the existing systems and interfaces as well as a lack of standardization still represent significant obstacles.

**Figure 3:**
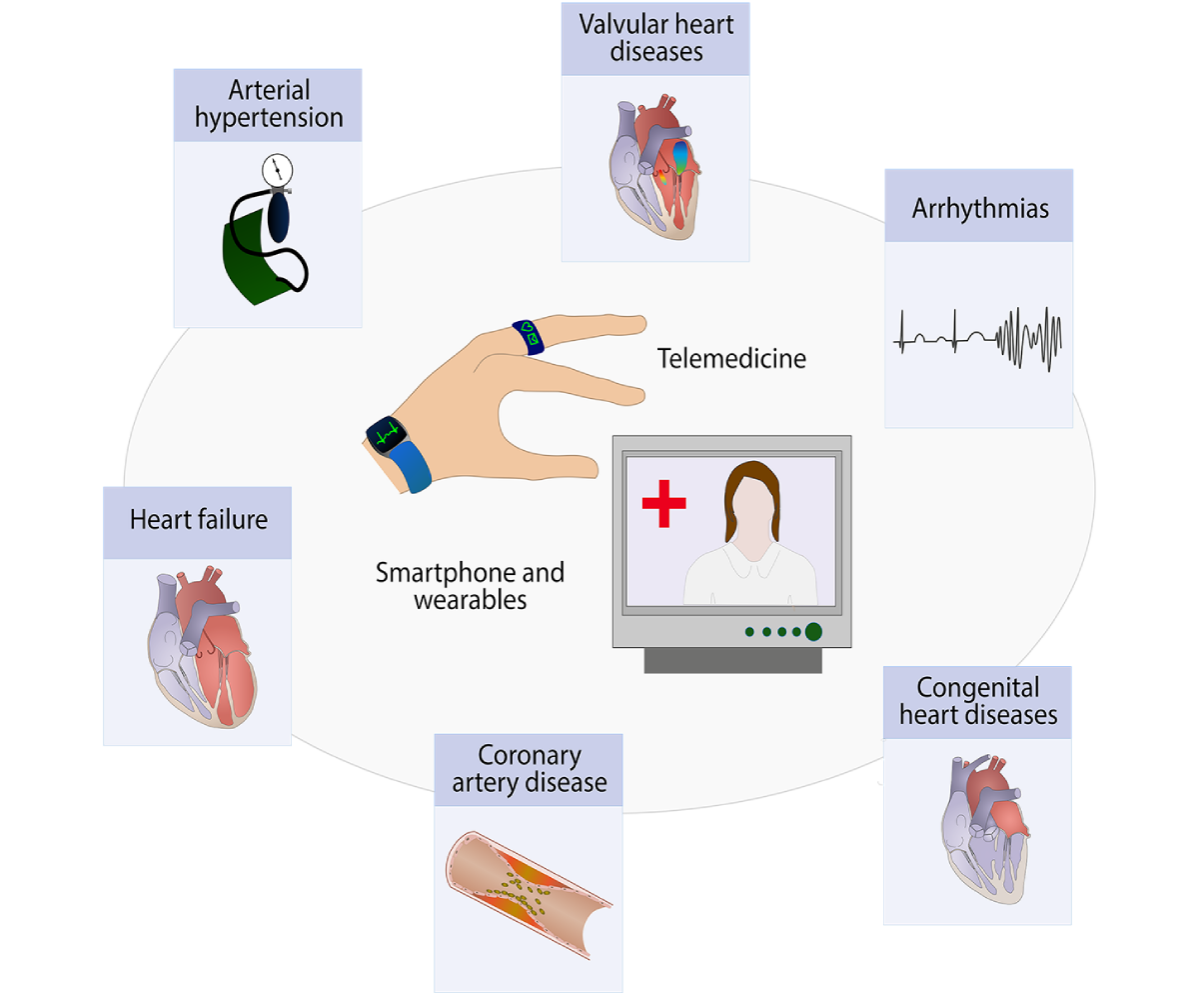
Digital medicine in cardiology.

This review provides an overview of all the present digital technologies in the cardiovascular field. This illustrates an enormous increase in knowledge and investigations in recent years with respect to optimized diagnostic and therapeutic strategies (Figure 3 and Table 2). We should not hesitate to face these opportunities and challenges in maintaining patient care at the highest possible standard. Thematic reappraisal, as previously described, is indispensable. Today, we are in a comfortable position to actively restructure and optimize our daily medical work.

**Table 2:** Diagnostic and therapeutic use of digital technologies^a^.

	Diagnostics		Therapy						
	Remote diagnosis	Events detection	Medication plan	Patient education	Remote rehabilitation	Training control	Prevent rehospitalization	Mortality	Costs
Heart failure	−	++	+++	++	++	++	++	+	−
Hypertension	+++	++	++	++	+	−	+	−	+
CAD^b^	+	++	+	++	+++	++	++	+	+
Rhythm^c^	+++	+++	+	+	−	+	+	−	+
CHD^d^	+++	−	−	−	−	++	−	+	+
VHD^e^	++	+	−	−	−	−	−	−	−

^a^+++, ++, and + indicate the degree of scientific evidence for positive effects on patient care, whereas − indicates negative results or missing data.

^b^CAD: coronary artery disease.

^c^Rhythm refers to arrhythmias.

^d^CHD: congenital heart disease.

^e^VHD: valvular heart disease.

Acute disease patterns such as myocardial infarction or sudden-onset arrhythmias can be documented via wearables and made accessible to remote telemedical diagnostics. With these modern methods, we have the unique opportunity to record short-term health conditions on demand or, as in the case of myocardial infarction, to save important time in the emergency cascade and thus reduce mortality.

The second major domain of digital technology in CVDs is related to diagnostics. Currently, it is possible to consult highly specialized medical experts for all types of questions regardless of the location. This was also the origin of digital technology in the cardiovascular field in the 1990s, when congenital heart defects were diagnosed via telemedicine expert consultations including guided echocardiography (Table 1). These remote diagnostic methods have been extended to many other cardiac pathologies and enable a very high medical standard even in rural areas or under the COVID-19–related restrictions.

In terms of cardiovascular therapy, digital technologies are expected to provide significant changes. Chronic diseases such as heart failure and arterial hypertension and secondary prophylaxis in coronary heart disease have huge health economic importance. Due to their high mortality, regular medical contact is essential for checking vital signs, adjusting medications, and detecting possible deterioration. Telemedicine offers a unique opportunity to replace these visits, at least in part, with digital consultations. Although immediate personal contact is certainly important and lost to some extent by telemedicine, more attention can be paid to the individual patient, travel time is reduced, and more patients can be treated by an individual physician. Detailed data records from wearables can be evaluated in a partially automated manner, highly up-to-date information about one’s own illness can be made available, medication plans can be managed and adjusted digitally, and methods of artificial intelligence can be used to optimize treatment strategies. The transitional phases from inpatient to outpatient care or to rehabilitation therapy can also be decisively supported by digital systems. Patients report not only an improved quality of life but also better performance, although it is possible to register any early recurrences of diseases or other adverse events in a timely manner.

Nevertheless, the possible risk of a decline in routine personal visits or the complete avoidance of physician contact may also have negative effects and potentially harm the patient. In an individual case, a human second assessment of the digital diagnostics remains essential and beneficial, especially for evaluating potential erroneous conclusions of digital algorithms.

Finally, both the patient and the treating physician must be aware that a considerable amount of sensitive patient data are made available to companies involved in digital medicine. As long as the product is not medically or scientifically approved, protected and sensitive handling of patient data is not guaranteed, as opposed to insurance companies or medical institutions.

In terms of evidence-based facts, heart failure is the best-investigated field in digital cardiovascular research. Proper patient selection provided by telemedicine monitoring systems has been shown to cause a slight but significant reduction in hospitalization and mortality [17,18]. Moreover, the 30-day rehospitalization rate of patients with heart failure was significantly reduced from 24% to 17% to 18% [50,51]. Evidence for arterial hypertension programs is limited, but initial meta-analyses have shown an additional reduction in systolic blood pressure by approximately 4 mm Hg [91,92]. In the group of coronary artery disease, remote diagnostics and telemedicine ECG transmission have proven the potential to reduce the door-to-balloon time by up to 30 minutes in acute myocardial infarction; this has direct implications on mortality due to reduced cardiac ischemia time [111,112]. Telemedicine offers optimized diagnostics in 3 other cardiovascular domains, including arrhythmias, congenital heart diseases, and valvular heart diseases, whereas its effects on mortality have not been proven in larger trials so far.

### Conclusions

Digitalization in cardiovascular medicine will certainly continue to gain importance in the near future, and it will be crucial to make the best possible use of these technologies. Today, the field of digital cardiovascular medicine has a strong focus on telemedicine technologies, as a lack of interoperability and standardization still limits applications such as automation and intelligent decision support systems, as well as modern medical IT-based research tools. Continual efforts will not only improve patient care but also have significant health economic saving potential for various CVD patterns. It is therefore important not to cling obsessively to old systems but to embrace innovations and actively help to shape them.

## Appendix

Multimedia Appendix 1 Overview of randomized controlled trials on heart failure.

## Abbreviations

AMULET: A New Model of Medical Care With Use of Modern Methods of Non-invasive Clinical Assessment and Telemedicine in Patients With Heart Failure
BEAT-HF: Baroreflex Activation Therapy for Heart Failure
CHAMPION: CardioMEMS Heart Sensor Allows Monitoring of Pressure to Improve Outcomes in New York Heart Association Class III Heart Failure Patients
CVD: cardiovascular disease
ECG: electrocardiogram
EVOLVO: Evolution of Management Strategies of Heart Failure Patients With Implantable Defibrillators
IN-TIME: Influence of Home Monitoring on Mortality and Morbidity in Heart Failure Patients With Impaired Left Ventricular Function
PRISMA: Preferred Reporting Items for Systematic Reviews and Meta-Analyses
REM-HF: Remote Management of Heart Failure Using Implanted Electronic Devices
Tele-HF: Telemonitoring to Improve Heart Failure Outcomes
TIM-HF: Telemedical Interventional Monitoring in Heart Failure
