# Supplementary material for: Digital Transformation in the Diagnostics and Therapy of Cardiovascular Diseases: Comprehensive Literature Review
Source: JMIR Cardio. 2023 Aug 30;7:e44983. doi: 10.2196/44983 (PMC10500361; doi:10.2196/44983)
Supplement: Multimedia Appendix 1 [file cardio_v7i1e44983_app1.docx]

**Supplementary Table 1: Overview on randomized controlled trials on heart failure**

| **Publication** | **Topic** | **Intervention** | **Primary endpoint** | **N** | **Follow-up** | **Result (Intervention vs. Control)** |
| --- | --- | --- | --- | --- | --- | --- |
| **IMPLANTED CARDIAC DEVICES** | | | | | | |
| Böhm et al, 2016 [21] | Fluid status alerts in ICD HF patients (OptiLinkHF) | Telemedical contact vs. usual care after fluid status alert | All-cause death and cardiovascular hospitalization | 1002 | 1.9 years | 45.0 vs. 48.1%; HR 0.87 (CI 0.72, 1.04; p=0.13) |
| Boriani et al, 2017 [27] | Monitoring of resynchronization devices (MORE-CARE) | Remote care (follow-up and alters) vs. usual care | Delay between alert and clinical decision | 154 | 1 year | **2 (IQR 1, 4) vs. 29 (IQR 3, 51; p=0.004) days** |
| Guedon-Moreau et al, 2014 [25] | Costs of remote monitoring vs. ambulatory ICD follow-ups (ECOST) | Visit every 12 months + alerts vs. visit every 6 months | Costs | 310 | 2.3 years | **1695 (±1131)€ vs. 1952 (±1023)€ (p=0.04)** |
| Lüthje et al, 2015 [28] | Remote monitoring and fluid management for ICD patients | Remote monitoring + alerts vs. usual care | HF-related hospitalizations | 176 | 1.3 years | Hospitalization HR 1.23 (CI 0.62, 2.44)  2EP: no differences for ICD shocks or mortality |
| Abraham et al, 2011 [29] | Wireless PA pressure monitoring in HF patients (CHAMPION) | PA pressure-guided management vs. usual care | Hospitalization | 550 | 1.3 years | **HR 0·70 (CI 0.60, 0.84; p<0·0001)** |
| Varma et al, 2021 [26] | Remote hemodynamic-guided therapy in CRT HF patients with a CardioMEMS PA pressure sensor | PA pressure-guided management vs. usual care | Hospitalization, PA pressure, medication, QoL | 190 | 1.5 years | **Hospitalization:  HR 0.70 (CI, 0.51, 0.96; p=0.028);**  **PA pressure: AUC -413.2±123.5 vs. 60.1±88.0 (p=0.002); medication titrations: 847 vs. 346 (p<0.001); QoL improvement: -13.5±23 vs. -4.9±24.8 (p=0.006)** |
| Landolina et al, 2012 [23] | Remote monitoring in ICD HF patients (EVOLVO) | Remote monitoring vs. usual care | Unplanned hospitalizations | 200 | 1.3 years | **59% vs. 93% events per year (p=0.005)** |
| Morgan et al, 2017 [22] | Remote monitoring for HF patients with implanted cardiac devices (REM-HF) | Remote monitoring vs. usual care | All-cause death and cardiovascular hospitalization | 1650 | 2.8 years | HR 1.01 (CI 0.87, 1.18; p=0.87) |
| Hindricks et al, 2014 [24] | Implant-based telemonitoring in HF patients (IN-TIME) | Telemonitoring vs. usual care | All-cause death, hospitalization, change in NYHA class, change in patient global self-assessment | 664 | 1 year | **OR 0·63 (CI 0.43, 0.90; p=0.013)** |
| **TELEMEDICINE** | | | | | | |
| Frederix et al, 2019 [30] | Telemonitoring and management of HF (TEMA-HF) | 6 months telemonitoring vs. usual care | All-cause mortality | 160 | 6.6 years | HR 0.83 (CI 0.57, 1.20; p=0.32);  2EP: less days lost due to HF; no difference in costs |
| Gingele et al, 2019 [31] | Effect of telemonitoring on functional status and quality of life in HF patients | Tailored telemonitoring vs. usual care | METS | 382 | 1 year | **Regression coefficient 0.318; p=0.01);**  2EP: QoL unchanged |
| Koehler et al, 2012 [16] | Telemedical interventional monitoring in HF (TIM-HF) | Remote telemedical management vs. usual care | All-cause mortality | 710 | 1 year | HR 0.97 (CI 0.67, 1.41; p=0.77) |
| Koehler et al, 2018 [18] | Telemedical interventional monitoring in HF2 (TIM-HF2) | Remote telemedical management vs. usual care | All-cause death and cardiovascular hospitalization | 1571 | 1 year | **4.88% vs. 6.64%, HR 0.80 (CI 0.65, 1.00; p=0.046)** |
| Piotrowicz et al, 2020 [32] | QoL after telerehabilitation for HF patients (TELEREH-HF) | Telerehabilitation vs. usual care | Extrahospital days | 850 | 2 years | 91.9 (±19.3) vs. 92.8 (±18.3) days; p=0.74 |
| Rahimi et al, 2020 [33] | Home monitoring with and without technology-supported management in HF | Digital home monitoring ± specialist support | Relative adherence to guideline-recommended therapy | 202 | 0.5 years | 0.54 (0.46, 0.62) vs. 0.61 (0.52, 0.70); p=0.25 |
| Ali et al, 2021 [34] | Person-centered care (digital platform and telephone support) in COPD and HF | Person-centered care vs. usual care | Composite EP: Self-efficacy, all-cause death and hospitalization | 222 | 6 months | No significant changes, p=0.47 |
| Ding et al, 2020 [35] | Effect of telemonitoring on compliance in HF (ITEC-HF) | Telemonitoring enhanced care vs. usual care | Relative compliance to weighing at least 4 days a week | 184 | 6 months | 74% vs. 60%, p=0.06 |
| Goldstein et al, 2014 [19] | Telemedicine medication reminder in HF | Electronic pill box vs. smartphone reminder vs. usual care | Medication adherence | 60 | 1 month | No significant changes, p=0.87 |
| Indrartna et al, 2022 [36] | Smartphone-based care for hospital to community transition | Teleclinical care vs. usual care | Unplanned readmission | 164 | 6 months | **No difference for 30-days readmissions (p=0.97), overall readmission 21/81 vs. 41/83 (p=0.02)** |
| Jimenez-Marrero et al, 2020 [37] | Telemedical care in HF patients with a LVEF≥40% | Telemedicine vs. usual care | Non-fatal HF events  2EP: HF care-related costs | 116 | 6 months | **22 vs. 56%; HR 0.33 (CI 0.17, 0.64; p<0.001)**  **2EP: 8163 vs 4993€; p=0.001)** |
| Pekmezaris et al, 2019 [38] | Telehealth self-management in underserved black and hispanic HF patients | Telehealth self-management vs. usual care | QoL and hospitalization | 104 | 3 months | No significant changes |
| Sahlin et al, 2022 [39] | Self-care management in HF (SMART-HF) | Home-based mobile tool vs. usual care | Self-care behavior scale  2EP: in-hospital days | 118 | 8 months | **21.5 (IQR 13.25, 28) vs. 26 (18, 29.75); p=0.014**  **2EP: 2.2 days less, RR 0.48 (CI 0.32, 0.74; p=0.001)** |
| Spaeder et al, 2006 [20] | Telemedical carvedilol titration in HF patients | Telemedical vs. usual care titration | Final daily dose and time to reach final dose | 49 | 3 months | **Final dose: 36.2 vs. 39.4 mg/d, p=0.52);**  **time: 33.6 vs. 63.7 days (p=<0.0001)** |
| Wagenaar et al, 2019 [40] | Effectiveness of ESC website and e-health on HF self-care (e-Vita HF) | Website vs. e-health vs. usual care | Self-care behavior scale | 450 | 1 year | 73.5 vs. 78.2 vs 70.8 after 3 months, no significant changes after 12 months |
| Krzesinski et al, 2022 [17] | Nurse-led telemedical support in HF patients (AMULET) | Nurse-led telemedical support vs. usual care | Cardiovascular death or unplanned HF hospitalisation | 605 | 1 year | **HR 0.69 (CI 0.48, 0.99; p=0.044)** |
| Ong et al, 2016 [41] | Remote monitoring of HF patients after discharge (BEAT-HF) | Nurse-led telemedical support vs. usual care | Rehospitalization within 6 months | 1437 | 6 months | HR 1.03 (CI 0.88, 1.20; p=0.74) |
| Chaudhry et al, 2010 [15] | Telemonitoring in patients with HF (TELE-HF) | Telemonitoring vs. usual care | All-cause death, hospitalization | 1653 | 6 months | 52.3 vs. 51.5% (p=0.75) |
| Cleland et al, 2005 [14] | Non-invasive telemonitoring for high-risk HF patients (TEN-HMS) | Telemonitoring vs. nurse telephone support vs. usual care | Proportion of days dead or hospitalized | 426 | 8 months | No significant changes (19.5 vs. 15.9 vs. 12.7%) |

CI, confidence interval; COPD, chronic obstructive pulmonary disease; CRT, cardiac resynchronization therapy; HF, heart failure; HR hazard ratio; ICD, implanted cardioverter defibrillator; IQR, interquartile range; LVEF, left ventricular ejection fraction; OR, odds ratio; PA, pulmonary artery; QoL, quality of life; 2EP, secondary endpoint.
